# On the simple random-walk models of ion-channel gate dynamics reflecting long-term memory

**DOI:** 10.1007/s00249-012-0806-8

**Published:** 2012-04-07

**Authors:** Agata Wawrzkiewicz, Krzysztof Pawelek, Przemyslaw Borys, Beata Dworakowska, Zbigniew J. Grzywna

**Affiliations:** 1 Department of Physical Chemistry and Technology of Polymers, Section of Physics and Applied Mathematics, Silesian University of Technology, Ks. M. Strzody 9, 44-100 Gliwice, Poland; 2 Division of Biophysics, Department of Physics, Warsaw University of Life Sciences—SGGW, Nowoursynowska 166, 02-787 Warsaw, Poland

**Keywords:** Random walk process, Conformational diffusion, Hurst analysis, BK channels, Activation gate

## Abstract

Several approaches to ion-channel gating modelling have been proposed. Although many models describe the dwell-time distributions correctly, they are incapable of predicting and explaining the long-term correlations between the lengths of adjacent openings and closings of a channel. In this paper we propose two simple random-walk models of the gating dynamics of voltage and Ca^2+^-activated potassium channels which qualitatively reproduce the dwell-time distributions, and describe the experimentally observed long-term memory quite well. Biological interpretation of both models is presented. In particular, the origin of the correlations is associated with fluctuations of channel mass density. The long-term memory effect, as measured by Hurst R/S analysis of experimental single-channel patch-clamp recordings, is close to the behaviour predicted by our models. The flexibility of the models enables their use as templates for other types of ion channel.

## Introduction

### Theoretical background

Potassium channels are integral proteins that enable rapid, selective transport of K^+^ ions across the cell membrane down their electrochemical gradient (Chung et al. 2007). In general, ion channels are not just simple pores with a constant permeability—they undergo conformational changes resulting in transitions between the conducting and non-conducting (open and closed) states. The probabilities of these states depend on channel-specific gating stimuli, of which the most common are membrane potential, ligand binding, and mechanical force (Hille 2001).

Ion-channel proteins comprise several subunits with different functions. In particular, distinct structural domains are responsible for the stimulus sensing and for the channel activation. Thus, stimulus–response relationships require a cooperative interaction between the sensor domains and the gate. When a gating stimulus occurs at the sensor domain, the conformational change is conveyed to the activation gate and changes the transition probability between the open and closed states.

Voltage-dependent, calcium-activated potassium channels (BK) are characterized by a large single-channel conductance (~100–300 pS) (Latorre and Miller 1983; Latorre et al. 1989; Marty 1983) compared with that of other potassium channels. These channels, which can be activated by membrane depolarization or by elevation of intracellular calcium concentration, can be found in many cells and tissues, for example neurons, chromaffin cells, the inner hair cells of cochlea, and muscles, and have an important function in several physiological processes (Cui et al. 2008). Irrespective of their unusually large conductance, BK channels remain highly selective for K^+^ ions over other cations (Cui et al. 2008).

In general, the putative structure of BK channels shares many similarities with that of voltage-dependent Kv channels (Ma et al. 2006; Liu et al. 2008a, b) and ligand gated potassium channels, for example MthK (Latorre and Brauchi 2006; Jiang et al. 2001, 2002; Shi et al. 2002; Xia et al. 2002; Tang et al. 2003; Yusifov et al. 2008; Kim et al. 2006, 2008). BK channels are tetramers of a channel protein encoded by the Slo1 gene, composed of seven transmembrane domains (S0–S6) and four cytosolic hydrophobic C-terminal domains (S7–S10) (Cox 2006; Cui et al. 2008; Latorre and Brauchi 2006). Functionally, segments S1–S4 form a voltage sensor domain (VSD), and segments S5–S6 form a pore-gate domain (PGD). The large cytoplasmic C-terminal domain serves as the primary ligand sensor. S0 is a segment that is absent from Kv and MthK channels and causes some problems in homology modelling, directing the N-terminus to the extracellular side of the membrane (Cui et al. 2008).

The simplest model of the channel’s gating kinetics can be described by a two-state system:

1 $$ \begin{array}{*{20}c} C & {\begin{array}{*{20}c} {k_{\text{O}} } \\ \to \\ \leftarrow \\ {k_{\text{C}} } \\ \end{array} } & O \\ \end{array} $$

in which the states of the channel switch randomly between the open and closed conformations with gating-factor-dependent kinetic rate constants (*k*_O_, *k*_C_). This kind of description may belong to a Markov class of model, if the state transition probabilities depend only on the current state of the system (Fuliński et al. 1998).

Invention of the patch-clamp technique (Sakmann and Neher 1995) has enabled experimental observation of the ionic currents at a single-channel level, which gave the opportunity to obtain the probability densities of closed *f*_C_(*t*) and open *f*_O_(*t*) dwell time intervals. For many channels, the open dwell-time distribution can be reasonably approximated by a single exponential function, but the closed dwell-time distribution fits better to the sum of many exponentials of the form (Goychuk and Hänggi 2002, 2003):

2 $$ f_{C} (t) = \sum\limits_{i = 1}^{N} {c_{i} \lambda_{i} \exp ( - \lambda_{i} t)} , $$

with weight coefficients obeying:

3 $$ \sum\limits_{i = 1}^{N} {c_{i} = 1} , $$

Quite often Eq. (2) can be replaced by a single stretched exponential (Millhauser et al. 1988) or a power law function (Sansom et al. 1989; Blatz and Magleby 1986; Ring 1986; Mercik and Weron 2001; Läuger 1988; Condat and Jäckle 1989). When the dwell time distribution can be described by a sum of exponentials, Markov models with either a few or many open and closed states have been proposed. Such models assume the channel protein to be found in a number of discrete states, separated by relatively high potential barriers (Sansom et al. 1989).

When multiple open and closed states are fitted to the model, a possibility arises for allosteric activation of the channel, as shown for BK channels as an example in Horrigan et al. (1999), Horrigan and Aldrich (1999), and Rothberg and Magleby (1999). The HCA model proposed by Horrigan et al. (1999) and Horrigan and Aldrich (1999) is a classical, many-state Markov model of BK channel voltage-dependent gating. It reflects well the channel’s behaviour at different values of membrane potential in the absence of any calcium ions and agrees quantitatively with experimental results. A crucial feature of the HCA model is the allosteric mechanism of BK channel activation, which is different from the strict coupling proposed in the literature to describe gating of the Kv channel (an example of which is the Hodgkin–Huxley model) (Hille 2001). Whereas in the strict coupling of Kv channel activation voltage sensor movement opens a channel, this is not so in allosteric activation. In the allosteric model (of BK channels) neither of the sensors directly opens the gate but all of them affect the gate, and each other, in an allosteric manner, and gate opening can even precede the sensor’s activation (Rothberg and Magleby 1999).

The calcium activation of BK channels is characterized in the voltage-dependent Monod Wyman Changeux (VD-MWC) model (Cox et al. 1997). Analogously to the previous case, because of incorporation of an allosteric mechanism, none of the Ca^2+^-activated sensor domains renders the channel conductive in a direct manner. Nevertheless, responding to the ligand binding on any of the multiple binding sites, the channel undergoes a conformational change that promotes the opening, and (extending the MWC formalism) indirectly alters the remaining binding sites by increasing the affinity for Ca^2+^. In this model, the open state has a higher Ca^2+^ affinity than a corresponding closed state with the same number of calcium ions bound. Thus, an open Ca^2+^-bound state is energetically preferred over a closed Ca^2+^-bound state. The voltage dependence of the gating is embedded in the equilibrium constants between the closed and open states.

Advanced approaches based on the HCA and MWC models have been proposed in the literature to account for both allosteric activation processes in a single model (Rothberg and Magleby 2000; Horrigan and Aldrich 2002). By extending the assumptions of its templates, these models reach a large number of states (e.g. 50-state two-tiered model); their purpose is to give an even more complete picture of the gating process over a wide range of voltages and calcium concentrations, assuming all the rate constants are correctly determined (which is not easy). What is important here it is that coupling between different functional parts of the channel may be strong, as for the sensor–gate interaction, or weak, as for the voltage sensor–calcium sensor interaction. An allosteric model allows the channel to open even in the absence of any voltage and calcium stimuli or to close when all the sensors are activated. In general, however, the greater the number of sensors activated the more energetically favoured an open conformation is. Both the voltage and the ligand sensors can switch from the resting to the activated state in either closed or open conformation of a channel.

The multi-exponential dwell-time distribution of the Markovian systems in some cases reduces to a single stretched exponential or a power law dependence. This observation leads to the introduction of the fractal (Liebovitch et al. 1987) and diffusive models (Millhauser et al. 1988; Läuger 1988; Condat and Jäckle 1989; Goychuk and Hänggi 2002, 2003) to address the voltage-dependent channels (Kv). According to these models, the channel protein exists in a continuous conformational space with a rather smooth energy surface, in contrast with a few, well separated discrete conformations determined by the potential wells, as expected from the finite state Markovian models.

A classical model of channel gating by a Markovian diffusion process over a very (infinitely) large number of similar energy closed states was introduced by Millhauser et al. (1988). This model produces the power law dependence in the dwell time distributions. It was generalised by Hänggi and Goychuk (2002, 2003) who investigated the gating dynamics as a continuous diffusion process with part of the closed state potential being voltage dependent. The model explains how the closed state dwell time distribution of a voltage dependent channel can change its character from a power law to an exponential function, depending on the membrane polarization. Moreover, the model justifies the exponential dependence of the Hodgkin–Huxley model used to describe the experimental relationship between the voltage and the rate of opening of a single gate in the potassium channels.

Further generalisations of diffusion modelling lead to non-Markovian conformational subdiffusion. It has been shown (Goychuk and Hänggi 2008) that the subdiffusive generalization reproduces the main features of the closed time distribution and predicts the autocorrelation function of the conductance fluctuations. The non-Markovian nature of the channel currents suggested by Fuliński et al. (1998) has also been addressed in different ways (Grzywna and Siwy 1997; Grzywna et al. 1999; Liebovitch and Sullivan 1987; Liebovitch et al. 1987). Interesting non-Markovian models were given by Grzywna and Siwy (1997) and Grzywna et al. (1999), who provide an alternative approach based on the assumption that the channel behaviour is governed by a nonlinear deterministic dynamics.

The models described capture many important features of the gating phenomena but not all of them. Among the missing features, a very interesting but not completely understood property of many channel species is the long-term memory, as seen by Hurst R/S analysis (Campos de Oliveira et al. 2006; Bandeira et al. 2008). The rescaled range analysis (R/S analysis, Hurst analysis; Hurst 1951; Hurst et al. 1965; Feder 1988; Borys 2011), when applied to the ionic current recordings, measures the correlation of adjacent dwell times of an ion channel (Varanda et al. 2000), commonly indicative of strong trend-reinforcing behaviour.

Whether the discrete Markovian models can produce a long-range memory was the subject of research (Varanda et al. 2000; Campos de Oliveira et al. 2006) on the 3, 4, and 11-state models. All the tests were negative. Whether Markovian models with more states can reproduce the long-range memory, as measured by the Hurst exponent, is, however, still an open question.

According to the experiments, the long-term memory in ion channel activity seems to be independent of external channel activating factors (Barbosa et al. 2007; Varanda et al. 2000 and the section “Analysis of experimental data” in this paper). It was observed that at each fixed voltage and calcium concentration (at a macro scale), and thus at each corresponding average sensor activation level (at a molecular scale), the long-term correlations in time series are of similar magnitude. It is, therefore, reasonable to link the memory with gate fluctuations under given conditions only. This point of view is also supported by the allosteric picture of BK channel gating dynamics, with gate fluctuations present even in the absence of gating sensor activation.

In this paper, we limit our considerations to gate fluctuations, ignoring the full HCA-MWC machinery; this has the advantage of showing the processes relevant to the long-term memory without obscuring them by the additional details of a gate–sensor coupling. For the gate dynamics we propose two simple random-walk models belonging to the diffusion class of models (Millhauser et al. 1988; Goychuk and Hänggi 2002, 2003). The models generate a dichotomous current time series as found in the experimental results (the patch-clamp recordings), and produce the long-term memory of the considered system. The open and closed-dwell time distributions evaluated for the simulated series are concurrent, at a qualitative level, with those observed experimentally. We also propose biophysical interpretations for both models.

### Description of the models and biological inspirations

It is stated in the “Theoretical background” that the long-term memory is independent of the sensor state. To check whether our models agree with such results, we need some simplified representation of biosystem activation. To propose a reasonable approach, we briefly review the ideas which are the basis of BK channel’s voltage and calcium sensor operation in relation to the gate.

The activation gate is a part of the channel protein responsible for blocking the ion flux through the pore (Chung et al. 2007). Because of studies on KcsA and MthK channels (Jiang et al. 2002) we have physical evidence of gate-open and gate-closed conformations of these potassium channels. A key difference between these is the bending of an inner helix segment at a glycine residue close to the selectivity filter, called the “Gly-hinge”.

In many potassium channels, the inner mouth of the pore (the channel’s entrance) is formed by the hydrophobic residues on the C-terminus of the inner helix (S6 in the canonical channel structure with six transmembrane segments). The residues are arranged in a bundle to block the potassium ion flux when the channel is closed. Because of the flexibility of the S6 domain at the Gly-hinge in the middle of S6 (sometimes assisted with the Pro-kinks several residues later), a gate-open conformation can be reached with inner helices well separated from each other (Jiang et al. 2002; Long et al. 2005; Ding et al. 2005; Cui et al. 2008). For the BK channels, it can be shown by mutations that the Gly-hinges are important but not mandatory structures to reach the open conformation, which contrasts with the Kv or MthK channels (Magidovich and Yifrach 2004). Experiments with quaternary ammonium ions show further that the BK channel’s selectivity filter entry is accessible in the gate-closed conformation, which suggests the existence of a secondary gate in the close vicinity of the selectivity filter, similarly to several other ligand-gated ion channels (Wilkens and Aldrich 2006; Flynn and Zagotta 2001; Bruenning-Wright et al. 2007; Cui et al. 2008).

The voltage sensor of the BK channel also differs significantly from the Kv’s, i.e. the gating charge is not confined to S4, but rather spreads over the whole voltage-sensing domain (VSD), and is much smaller than in Kv channels. Furthermore, the ligand sensor is not exactly similar to MthK, because the gating ring of a BK channel has important differences from that of the MthK one (e.g. the gating ring of MthK is formed by eight identical RCK domains, whereas in the BK channel it is formed by four RCK1 and four RCK2 domains).

Ligand sensor operation relies on the interaction between a C-terminal intracellular RCK domain which forms a gating ring which expands on calcium binding, and the inner helix connected to the RCK via an S7-residue linker, whose mutations reveal a profound effect on the gating dynamics (Jiang et al. 2002). The voltage dependency of the BK channels is hypothesized on the basis of homology with the Kv channel gating, which depends on the motion of the charged S4 segment binding through the S4–S5 linker to the S6 segment at a Pro-X-Pro motif, triggering mechanical translations of S6 which result in gating, probably via Gly-hinge bending (Ding et al. 2005).

In our approach, the conformational dynamics of the activation gate is described by a discrete random walk process performed by the RC (reaction coordinate) over the conformational space. We could assign the RC a meaning of, e.g., the hinge angle but, because the actual gating blocks of a BK channel are not yet known, we treat RC as abstract coordinate in the conformation space. The effect of the sensors on the gate is allosteric in nature in the BK channels and is introduced by a drift force acting on the reaction coordinate. Such a drift force approach to sensor activation retains the allosteric picture of the system, in which sensor activation is not mandatory for gate opening. Furthermore, the drift force is a good candidate to mimic sensor action, because the sensor seems to operate by forcing the Gly-hinge. In general, this drift force may depend nonlinearly on the reaction coordinate, calcium concentration, and voltage, $F_{\text{D}} = F_{\text{D}} ([{\text{Ca}}^{2 + } ],V,{\text{RC}}).$ In our case however, the actual transmission of the gating stimuli is irrelevant (we have assumed that the long-term memory is independent of these stimuli), and we have only checked whether the activation bias modifies the Hurst effect. Having no hints on the RC dependency of the drift force, it was assumed to be constant, which corresponds to the linear ramp potential.

In the diffusive modelling we can assume that the open and closed states of the gate embrace two manifolds of energetically similar substates. The manifolds observed in a macroscopic state can be associated with a set of single protein structure vectors, nearly equal in energy, which retain the macroscopically observed property of the state. From such a viewpoint, the shifting between two different macro-states is realized by a diffusion process between adjacent conformations.

Because we approximate the conformational dynamics in terms of the one-dimensional reaction coordinate *x*(*t*) in a conformational potential *U*(*x*), as was shown by (Goychuk and Hänggi 2002, 2003), the question which arises is, is this approximation valid for a large 3D system such as a protein? Research on protein folding suggests this may often not be true (Dill et al. 1995). Nevertheless, if we consider the activation gate as a hinge, movement of which is effectively restricted to one degree of freedom (or the other possible conformations that can be projected on it), the idea of a one-dimensional reaction coordinate may be valid.

**Fig. 1:**
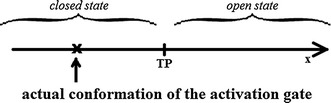
Schematic illustration of a channel gate’s possible state transitions. One can observe openings (C → O), closings (O → C), and changes among open (O → O) or closed (C → C) states. *TP* threshold point separating open and closed states, *x* reaction coordinate

The activation gate’s conformational space is illustrated in Fig. 1. It is divided into two parts, which correspond to the open and closed states of the gate, separated by a threshold point TP. The open and closed states can have different energies, which reflect the tendency of a channel to maintain a conducting or non-conducting state under fixed external conditions. The reaction coordinate performs a random walk in the conformation space with positions before the threshold point (TP) indicating a closed state and those behind the threshold point indicating an open state, as shown in Fig. 1.

**Fig. 2:**
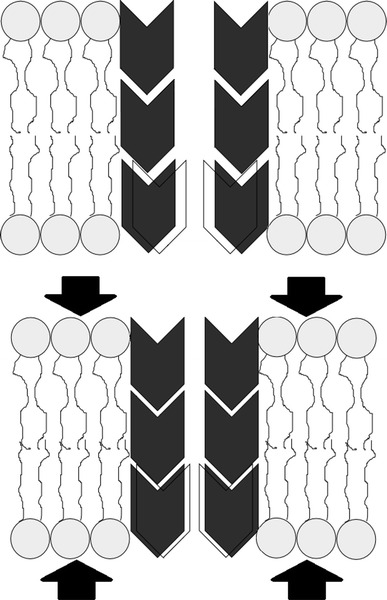
Schematic illustration of the changes of local membrane mass density and its effect on channel protein. The range of channel gate’s accessible conformations can be reduced or increased as a result of membrane squeezing and its relaxation, respectively, as denoted by the frame on the *lower* “diamond” in the figure

We propose that the long-term memory of the system may result from synchronized fluctuations of the activation gate conformational space boundaries (Model 1). The biophysics of such fluctuating boundaries may be explained by considering what happens after squeezing of the channel protein. This may originate from the local fluctuations in the membrane thickness near the channel location. Squeezing will increase the mass density of the channel protein (including the activation gate), and the atoms (being restricted by their Van der Waals’ volumes) have less “space” to move, reducing the number of energetically equivalent substates within a given macro-state. In other words, the boundaries of the conformational space move to reduce the maximum conformational difference between the substates in the closed and open conformations. The opposite may be observed in case of channel protein relaxation by the decrease in the local mass density. This behaviour is schematically depicted in Fig. 2.

Another way of introducing fluctuations of channel density to the gate dynamics is to add a synchronous drift force acting on the reaction coordinate (Model 2). In such picture, lateral compression or relaxation of the channel does not strictly limit the size of the conformational space, but rather produces a drift force acting on the reaction coordinate and favouring the occupancy of substates near the threshold (by relaxation) or near the boundaries (by compression). Such an approach reproduces the long-term memory of the channel system and enables the boundaries to be kept constant. It is interesting to notice that the effect of the compressive force is exactly opposite in Model 2 compared with Model 1. In Model 1, the compression favours rapid state switching whereas in Model 2 this regime is reached in a relaxed state. This may be important for experimental validation of these models. Model 2 drops the assumption of constant energy within the macro-state in the absence of gate–sensor coupling but energy variation among the substates is a smooth, slowly increasing or decreasing function of the RC (Fig. 5).

**Fig. 3:**
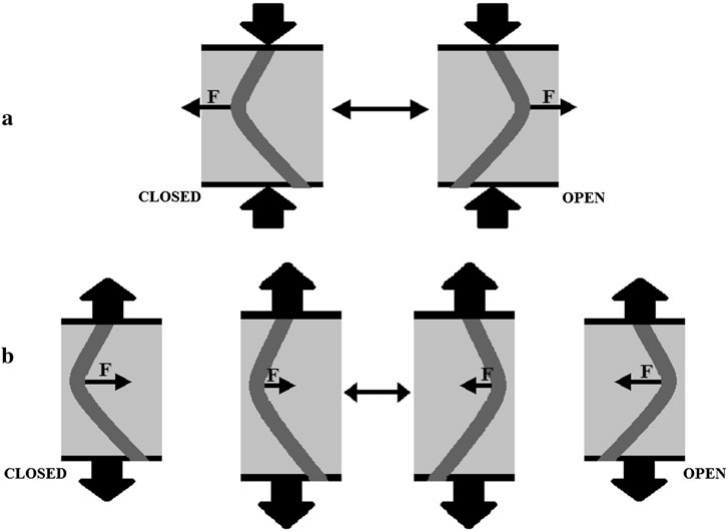
Squeezing of the membrane will generate a force acting on the channel segment, which is related to gating, and causing its bending. Bending motion may lead either to the gate’s closed or open conformations (**a**). Stretching of the membrane results in occurrence of a force which enables relaxation of the bent segment. The force strength depends on current thickness of membrane (and consequently, degree of bending) (**b**)

The conditions for Model 2 may occur if there is a protein segment, connected with gating, which has a large amount of space available for bending. In such case, reduction in the membrane thickness causes the segment to bend, i.e. a force is generated. If this bending can take place either toward or away from the open conformation, which is schematically shown in Fig. 3, then it can be described by the proposed model.

#### Model 1

**Fig. 4:**
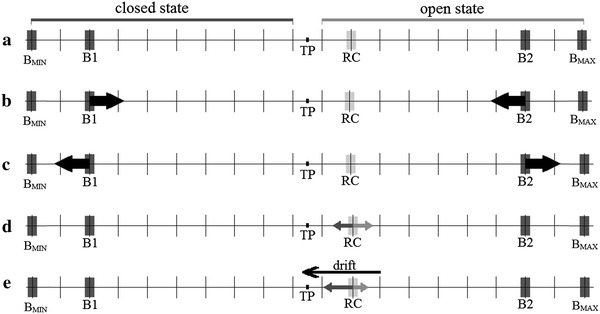
Schematic representation of Model 1. **a** The one-dimensional lattice has 20 nodes, among which first 10 represent closed states of the channel whereas the other nodes correspond to open states. Boundaries (*B*1, *B*) can move, simultaneously reducing (**b**) or increasing (**c**) the space accessible to the reaction coordinate (RC). Without any external force, no direction of motion is preferred (**d**), otherwise a drift in the direction down the potential energy gradient occurs (**e**), which changes the probability of finding the system in a given state

**Fig. 5:**
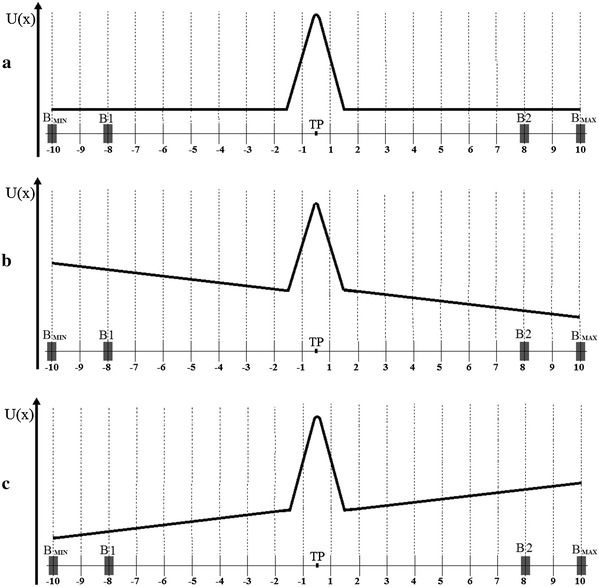
Schematic representation of the potential function associated with Model 1. **a** No force arising from sensor activity is acting, in particular, on the direction of the gate. The corresponding conformational potential energy *U*(*x*) of the reaction coordinate is “flat” and symmetric around the threshold point separating open and closed substates. If a nonzero drift force is present, it is represented by the conformational potential energy *U*(*x*) sketched on **b** or **c**. In **b** external potential field is expressed by a decreasing ramp function. As a result, open states will be preferred. Conversely, in a linearly increasing potential field closed states will be preferred (**c**)

The lattice variables of Model 1 are shown in Fig. 4. The conformational space of the gate was projected to one dimension and divided into two parts of equal length separated by a threshold point. The channel is closed if the reaction coordinate occupies the position below the threshold point, otherwise it is open. The transition between the open and the closed conformations requires overcoming of a transition potential barrier as shown on the energy landscape in Fig. 5a.

The range of possible reaction coordinate values in the conformational space is limited by the two moveable boundaries *B*1 and *B*2. The motion of *B*1 and *B*2 corresponds to thermal fluctuations in the membrane thickness and internal strains within the protein segments which restrict the conformational space of the closed and open states. The boundary fluctuations are synchronized in direction (e.g. when the conformational space of an open state shrinks, the same should happen to the conformational space of the closed state), and the probabilities of these increasing or reducing the accessible conformational space were set equal to 0.5.

Because the mass of the fluctuating membrane is much higher than the mass of the putative activation gate, the boundary fluctuations are realized on a larger time scale, i.e. if we assume that a change of the gate state is recognizable in one time step in simulation, then the change of boundaries may be performed after a given number of time steps according to the assumed relationship of the diffusion time scales:

4 $$ D_{\text{B}} = \frac{{D_{\text{RC}} }}{600}, $$

which results from fitting to the experimental data (*D*_B_ is the boundary diffusion coefficient and *D*_RC_ the reaction coordinate diffusion coefficient).

To test for the independence of the long-term correlations on sensor activation, a constant drift force from the channel sensors may be added to the system. The biassed random walk process can stand for the behaviour of a channel gate at a fixed value of the activating or inactivating stimulus. For example, in terms of a high value of the membrane potential (e.g. *V* = 80 mV) open substates are preferred, so the additional drift force will favour movement of the gate toward the open conformation. As a result, the energy structure of substates may change at different activation levels, as illustrated in Fig. 5b, c.

The probabilities of the value of the reaction coordinate (RC) decreasing (*q*) or increasing (*p*) were evaluated according to the formulas (Berg 1983):

5 $$ p = \frac{1}{2} - \frac{\Updelta U}{4kT}, $$

6 $$ q = \frac{1}{2} + \frac{\Updelta U}{4kT}, $$

where *k* is the Boltzmann constant, *T* denotes absolute temperature and Δ*U* is a potential energy difference within a lattice step centred around the reaction coordinate. The potential *U*(*x*) is postulated to take the following form (depicted in Fig. 5):

7 $$ \left\{ {\begin{array}{*{20}c} {\begin{array}{*{20}c} {U(x) = (x - B1) \cdot A + U_{{B1}} ;\quad x \in \langle B1;{\text{TP}} - 1.5)} \\ {U(x) = (x - ({\text{TP}} - 1.5)) \cdot B + U_{{{\text{TP}} - 1.5}} ;\quad x \in \langle {\text{TP}} - 1.5;{\text{TP}})} \\ {\begin{array}{*{20}c} {U(x) = (x - {\text{TP}}) \cdot ( - B) + U_{\text{TP}} ;\quad x \in \langle {\text{TP}};{\text{TP}} + 1.5\rangle } \\ {U(x) = (x - ({\text{TP}} + 1.5)) \cdot A + U_{{{\text{TP}} - 1.5}} ;\quad x \in ({\text{TP}} + 1.5;B2\rangle } \\ {A = \frac{{U_{{{\text{TP}} - 1.5}} - U_{{B1}} }}{{\text {TP}} - 1.5 - B1}} \\ \end{array} } \\ \end{array} } \\ {B = \frac{{U_{\text{TP}} - U_{{{\text{TP}} - 1.5}} }}{{\text {TP}} - ({\text {TP}} - 1.5)}} \\ \end{array} } \right., $$

where *B*1 and *B*2 are the locations of the left-hand and right-hand boundaries, respectively, TP is the threshold point position, *U*_*B*1_, *U*_TP_, and *U*_TP−1.5_ denote the potential values at given points, and *A* and *B* are the potential slopes between points *B*1 and TP − 1.5 and between points TP − 1.5 and TP, respectively. According to Eq. (7) the constant *A* represents the drift force toward the open or closed states of the system and the constant *B* describes the repulsive force originating from the potential barrier between the open and closed states.

The lattice size was set to 2*B*_MAX_ nodes, where *B*_MAX_ determines the maximum size of the open (and closed) state space. This space is further restricted by the reflecting boundary positions (*B*1, *B*2), which were initially set to −*B*_MAX_/2 and *B*_MAX_/2. The starting RC position was set to −1 (a closed substate nearest the threshold TR = 0). The time step length to move the reaction coordinate *t*_RC_ was equal to 1 and the time step length to move the boundaries was set to *t*_B_ = 600 (i.e. $D_{\text{B}} = \frac{{D_{\text{RC}} }}{600}$).

The model variables were estimated by the gradient optimization technique. The values were adjusted in the direction of decreasing error up to a point when the relative errors of the Hurst exponent (*E*_H_), the open state probability (*E*_pop_), and the open and closed state dwell time histograms fitting (*E*_dw,open_) and (*E*_dw,closed_) were less than 5 %, and their sum (*E*) was less than 10 %. The overall error was given by the formula:

8 $$ E = E_{\text{H}} + E_{\text{pop}} + E_{\text{dw,open}} + E_{\text{dw,closed}} $$

where:

9 $$ E_{\text{H}} = \frac{{\left| {H_{\exp } - H_{\text{sim}} } \right|}}{{H_{\exp } }}, $$

10 $$ E_{\text{pop}} = \frac{{\left| {p_{\text{op,exp}} - p_{\text{op,sim}} } \right|}}{{p_{\text{op,exp}} }}, $$

11 $$ E_{\text{dw,open}} = \frac{{\left| {\chi_{\text{exp,open}}^{2} - \chi_{\text{sim,open}}^{2} } \right|}}{{\chi_{{\exp ,{\text{open}}}}^{2} }}, $$

12 $$ E_{\text{dw,closed}} = \frac{{\left| {\chi_{{\exp ,{\text{closed}}}}^{2} - \chi_{\text{sim,closed}}^{2} } \right|}}{{\chi_{\text{exp,closed}}^{2} }}. $$

where *H*_exp_ is the Hurst exponent of the experimental series’ subsequent open and closed dwell times, *H*_sim_ is the Hurst exponent of a series of subsequent open and closed dwell times obtained from the simulated data, *p*_op,exp_ is the open state probability calculated from the experimental data, *p*_op,sim_ is the open state probability calculated from the simulated data, and *χ*_exp_ and *χ*_sim_ are obtained on the basis of an appropriate normalized dwell time histogram. *χ*_exp_ is calculated according to the formula:

13 $$ \chi_{\exp }^{2} = \frac{1}{N}\sum\limits_{i = 1}^{N} {\delta_{i}^{2} } $$

where *N* is the number of the dwell time histogram bins and *δ*_*i*_ is the standard deviation of a given bin (the standard deviation corresponds to the five different patch-clamp traces obtained under the same conditions of voltage and calcium concentration).

*χ*_sim_ is described by the equation below:

14 $$ \chi_{\text{sim}}^{2} = \frac{1}{N}\sum\limits_{i = 1}^{N} {(p_{i,\exp } - p_{{i,{\text{sim}}}} )^{2} } $$

where *N* is the number of the dwell time histogram bins and *p*_*i*_ is the probability corresponding to the *i*th histogram bin.

The sensitivity of the total error (*E*) to the terms of Model 1 is given in Appendix 3.

The optimum values of the terms in Model 1 are presented in Table 1.

**Table 1:** Values of the terms used in the Model 1 simulation

Term	Value
*B*_MAX_	14 rcu
*B*_MIN_	−14 rcu
TP	0 rcu
Reaction coordinate diffusion coefficient *D*_RC_	10^4^ rcu^2^/s
Barriers diffusion coefficient *D*_B_	*D*_RC_/600
*U*_TP_ − *U*_TP−1.5_	1.0 kT
Initial position of RC	−1 rcu
Initial position of *B*1, *B*2	Floor (*B*_MIN_/2), Floor (*B*_MAX_/2) rcu^a^

^a^ *rcu* reaction coordinate unit

The simulation details of Model 1 (and Model 2) are summarized in Appendix 2.

#### Model 2

**Fig. 6:**
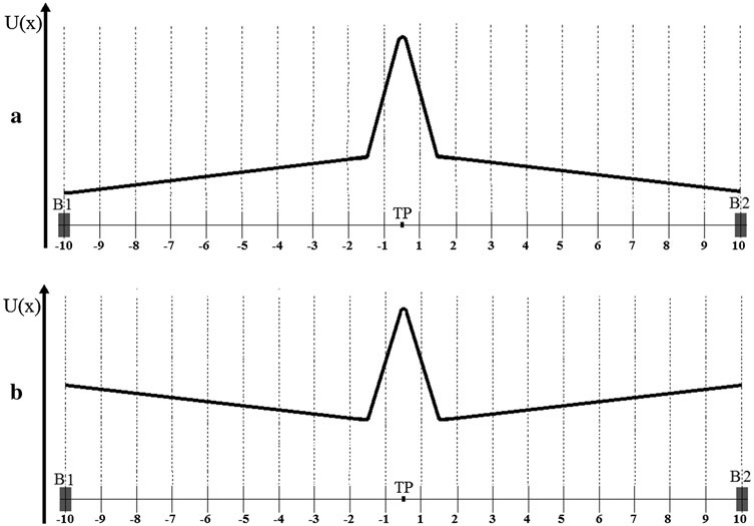
Schematic representation of the potential function associated with Model 2. **a** The random drift force facilitates movement toward boundaries. As a result, the conformational potential energy *U*(*x*) of the reaction coordinate increases from the boundary *B*1 to the threshold point TP and decreases from TP to the second boundary *B*2. In the opposite case, because of the effect of random force, positions around the threshold are preferred. The appropriate potential energy *U*(*x*) is sketched in **b**

Model 2 differs in the way the channel protein density fluctuations are introduced to the gate dynamics. The boundary positions (*B*1, *B*2) are kept constant, and additional, random drift force is added to the system which makes synchronized changes to the energy structure of the open and closed states, as presented in Fig. 6. This force causes the reaction coordinate to fluctuate near the threshold point (TP) or conversely, far away from it. The force fluctuations follow their own random walk, which is slower than the gating dynamics. The appropriate potential is depicted in Fig. 6 and is given by:

15 $$  \left\{ {\begin{array}{*{20}c} {\begin{array}{*{20}c} {U(x)= (x - B1) \cdot A + U_{B1} ;\quad x \in\langle B1;{\text{TP}} - 1.5)} \\ {U(x) = (x -({\text{TP}} - 1.5)) \cdot B + U_{{{\text{TP}} - 1.5}} ;\quad x \in \langle {\text{TP}} - 1.5;{\text{TP}})} \\{\begin{array}{*{20}c} {U(x) = (x - {\text{TP}}) \cdot ( - B)+ U_{\text{TP}} ;\quad x \in \langle {\text{TP}};{\text{TP}} +1.5\rangle } \\ {U(x) = (x - ({\text{TP}} + 1.5)) \cdot ( - A)+ U_{{{\text{TP}} - 1.5}} ;\quad x \in ({\text{TP}} +1.5;B2\rangle} \\ {A = \frac{{U_{{{\text{TP}} -1.5}} - U_{B1} }}{{{\rm TP} - 1.5 - B1}}} \\\end{array} } \\ \end{array} } \\ {B = \frac{{U_{\text{TP}} -U_{{{\text{TP}} - 1.5}} }}{{{\text{TP}} - ({\text{TP}} -1.5)}}} \\ \end{array} } \right.,  $$

where *B*1 and *B*2 are the locations of the left-hand and right-hand boundaries, respectively, TP is the threshold point position, *U*_*B*1_, *U*_TP_, and *U*_TP−1.5_ denote the potential values in given points, and *A* and *B* are the potential slopes between points *B*1 and TP − 1.5, and between points TP − 1.5, and TP, respectively. The *A* constant represents the drift force which facilitates the RC locations near the threshold point or near the boundaries. The *B* constant describes the repulsive force originating from the potential barrier between open and closed states.

**Fig. 7:**
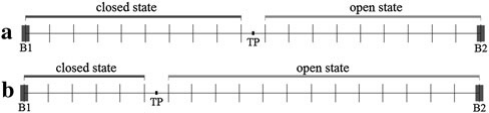
Schematic representation of the diffusive space used in Model 2. Boundaries (*B*1, *B*2) and the threshold position (TP) are fixed during the simulation. **a** If no gating stimulus is present in the system, the manifolds of closed and open states are of the same length. **b** The different numbers of possible open and closed substates account for preference of the channel for a particular macroscopic state. Among 20 nodes of the lattice used as the diffusive space first six indicate the closed substates of the gate, the other 14 nodes indicate open substates

The effect of the sensors on gate operation is reflected by the threshold point location. The conformational space is divided into two parts not necessarily equal, and the TP position is chosen such that the probability of finding the gate in an open state resembles the channel open probability under the given experimental conditions (Fig. 7). Such a mechanism can be justified by considering the effect of the sensing domains (e.g. the S5–S6 linker) on the gate, where they move the part of protein responsible for gating to increase the number of accessible closed conformations and reduce the number of open ones, or the opposite. The allosteric picture of the channel’s activation is conserved within such approach.

**Fig. 8:**
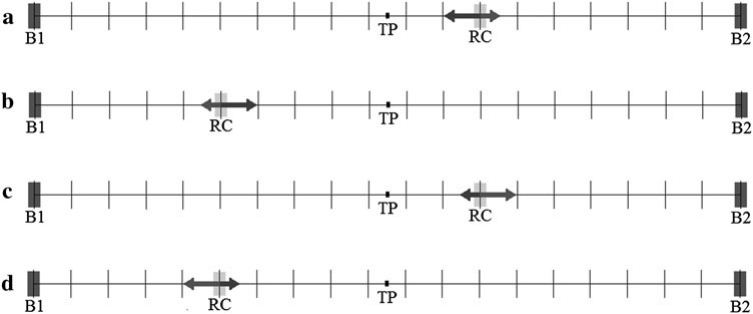
Schematic representation of Model 2. The reaction coordinate (RC) shifts either toward the threshold position (TP) with probability *p*_T_ represented as an *arrow* pointing toward TP (**a**, **b**, *p*_T_ > *q*_T_) or away from it (*arrow* pointing toward *B*1 or *B*2) (**c**, **d**, *q*_T_ > *p*_T_). The potential slope and, consequently, the values of *p*_T_ and *q*_T_ evolve randomly during the simulation (at a slower rate than evolution of the reaction coordinate)

The jump probabilities to the threshold point (*p*_T_), and to the boundaries (*q*_T_), are evaluated by use of Eqs. (5) and (6) (with *p*_T_ = *p* and *q*_T_ = *q* when RC < TP, and *p*_T_ = *q* and *q*_T_ = *p* when RC > TP), and the potential given by Eq. (15). The random walk performed by the RC is illustrated in Fig. 8.

The landscape potential slope (the drift force):

16 $$ F_{\text d} = \pm \frac{{U_{{{\text {TP}} - 1.5}} - U_{B1} }}{{\text {TP}} - 1.5 - {B1}} $$

is controlled by a separate unbiased random walk of *U*_*B*1_, taking place in a time scale a factor of 1,200 slower than the time scale of the reaction coordinate. The potential slopes cannot exceed minimum and maximum values, as specified in Table 2, with the other simulation values.

**Table 2:** Values of the terms used in simulation of Model 2

Term	Value
*B*1	−18 rcu
*B*2	18 rcu
Reaction coordinate diffusion coefficient *D*_RC_	10^4^ rcu^2^/s
Drift force diffusion coefficient *D*_DF_	*D*_RC_/1,200
*U*_TP_ − *U*_TP−1.5_	0.2 kT
Initial position of RC	−1 rcu
Initial drift force	0.00 kT/rcu
Drift force’s increment |Δ*k*|	0.005 kT/rcu
Max value of *F*_d_	0.20 kT/rcu
Min value of *F*_d_	−0.20 kT/rcu

The model terms are estimated, analogously as for Model 1, by the gradient optimization technique.

## Materials and methods

### Experimental

#### Cell line and solutions

Human bronchial epithelial cells were cultured on Petri dishes in minimum essential Eagle’s medium (Sigma) with 10 % fetal calf serum, 100 units/ml penicillin, and 100 μg/ml (PAA) at 37 °C in 5 % CO_2_. For patch-clamp recordings, the culture medium was exchanged for an extracellular solution containing 4 mM CaCl_2_, 2 mM EGTA, 10 mM HEPES, and 135 mM potassium gluconate, pH 7.3.

#### Electrophysiology

Experimental results were recorded from outside-out patches of human bronchial epithelial cells. The measurements were performed at room-temperature (20–21 °C). In all experiments symmetrical solutions on either side of the cell membrane were used. Patch pipettes were pulled from borosilicate capillary tubes of 1.2 mm diameter (Clark Electromedical Instruments) on a Narishige micro puller. The tips of the pipettes were smoothed in a Narishige micro forge. The ion currents were recorded using an Axopatch 200B amplifier (Axon Instruments). The experimental data were low-pass filtered at 10 kHz and transferred to a computer at a sampling frequency of 20 kHz using Clampex 7 software (Axon Instruments). Single currents were initially analysed by use of pClamp 7 software (Axon Instruments). The single-channel recordings were analysed at a fixed pipette potential. The experiments were carried out at: −80, −60, −40, −20, 20, 40, 60, and 80 mV and five independent measurements were recorded for each voltage. Each experiment was performed on a different patch (so we obtained 8 × 5 = 40 different patch-clamp recordings in total). The channel current was measured at time intervals of Δ*t* = 5 × 10^−5^ s. The ionic current measurement error was Δ*I* = 5 × 10^−4 ^pA. Each of patch-clamp recordings lasted 300 s, thus the experimental time series comprised *N* = 6 × 10^6^ current values at the applied time resolution of the measurement.

### Data analysis

#### Event detection

Investigating experimental time series, we considered two modes of ion-channel conduction, here called the open (conducting) and closed (non-conducting) states. The threshold current value used to identify transitions between following states was evaluated by use of a procedure described elsewhere (Mercik et al. 1999). The ion current probability density function (PDF) approximated by the nonparametric kernel density estimate with the Epanechnikov kernel was plotted on a log–log scale. The resulting graph reflects the bimodal nature of the analysed data set. The estimate obtained may be regarded as a mixture of two unimodal densities that satisfy power laws. By means of linear regression we obtained the intervals in both component distributions where the power laws and the corresponding formulas expressing scaling relationships were valid. A point of intersection of the power law plots indicates a threshold current value, in relation to which we identify open and closed states.

It is well worth noticing that if one applies the double Gaussian method of threshold current determination, the difference between the values obtained and those estimated by the kernel density method is less than 5 % (for each patch-clamp time series obtained in our experiments). The nonparametric technique of the PDF approximation avoids the assumption that the analysed data belong to any particular distribution, so we prefer this approach.

#### The R/S Hurst analysis

The rescaled range analysis (R/S Hurst analysis) is an important statistical method used for testing whether a system under consideration has long-term memory (Hurst 1951; Varanda et al. 2000). The Hurst exponent (*H*) describes the time scaling of a range (*R*) in a random motion, normalized to zero mean increments, $R \propto t^{H} .$ It can take values from 0 to 1, and provides a means of classification of temporal series in terms of predictability. A Hurst exponent of 0.5 is indicative of purely random behaviour of the system, and is an easily derived characteristic of the scaling of the standard deviation in Brownian motion (Risken 1996) (it is also characteristic of the scaling of *R* in the Brownian motion, as shown with somewhat larger effort by Borys 2011). In such a case there is no correlation among any element of the temporal series. If 0 < *H* < 0.5 the system is said to be anti-persistent, i.e. the range of corresponding anomalous Brownian motion grows slower than randomly. This means that a positive increment will tend to be followed by a negative one (and vice versa). The time series with a Hurst exponent value from 0.5 to 1 is persistent, or trending, i.e. the range of the corresponding Brownian motion grows faster than randomly. This means that a positive increment will tend to be followed by a positive one and a negative increment will tend to be followed by a negative one. The larger the *H* value, the stronger the trend, and the easier to predict the future behaviour of the system. In this paper we carry out a Hurst analysis for a sequence of adjacent open and closed times for both the BK channel recordings available, and the modelled data.

The procedure for evaluating the Hurst exponent can be found in many references (Hurst 1951; Hurst et al. 1965; Feder 1988; Varanda et al. 2000; Borys 2011). Details are also included in Appendix 1.

## Results and discussion

### Analysis of experimental data

**Fig. 9:**
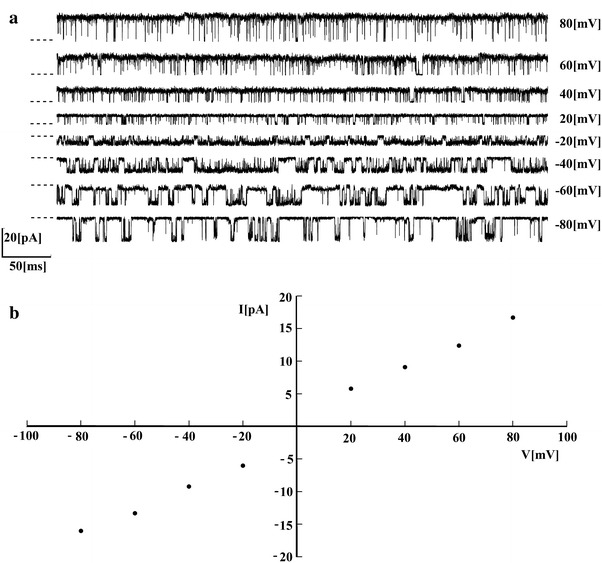
**a** Samples of the original ion current signal recorded from a single BK channel over the range of membrane potentials in fixed calcium concentration ([Ca^2+^] = 2 mM). *Dashed line* indicates closed state of the channel. **b** Current–voltage relationship for the BK channel recordings presented in **a**

The samples of original single-channel patch-clamp recordings, and the corresponding current–voltage relationship, are shown in Fig. 9. As one can see in Fig. 9a, channel opening varies with the potential value in a way typical of voltage-dependent ion channels, i.e. membrane depolarisation leads to an increase in the frequency of occurrence of the open state. According to Fig 9b, the reversal potential was near 0 mV. Under the experimental conditions, the mean conductance was estimated to be 235.6 pS.

Table 3 shows the values of the Hurst exponent, *H,* and the channel opening probability, *p*_o_, for the single-channel recordings obtained at different voltages *V* at fixed calcium concentration [Ca^2+^] equal to 2 mM. It reveals no significant effect of membrane depolarization on long-term correlations. There is just small variability around the mean of *H*, which is expected because *H* is a random variable with a standard deviation of 0.1 (Hurst et al. 1965). The mean *r* ^2^ coefficient of linear regression (used by evaluating *H*) for all experimental dwell time series was equal to 0.992.

**Table 3:** Mean experimental values of the Hurst exponent (*H*), the open state probability (*p*_o_), and mean open and closed dwell time (*Τ*_open_ and *Τ*_closed_) obtained at [Ca^2+^] = 2 mM under different voltage conditions

*V* (mV)	*H* ± Δ*H*	*p*_o_ ± Δ*p*_o_	*Τ*_open_ ± Δ*Τ*_open_ (ms)	*Τ*_closed_ ± Δ*Τ*_closed_ (ms)
−80	0.70 ± 0.05	0.17 ± 0.02	0.70 ± 0.10	0.46 ± 0.10
−60	0.76 ± 0.02	0.33 ± 0.07	1.00 ± 0.30	0.60 ± 0.20
−40	0.63 ± 0.03	0.63 ± 0.03	1.30 ± 0.15	0.80 ± 0.10
−20	0.73 ± 0.04	0.78 ± 0.07	1.20 ± 0.20	0.25 ± 0.10
20	0.82 ± 0.05	0.86 ± 0.07	1.90 ± 0.35	0.25 ± 0.10
40	0.63 ± 0.04	0.92 ± 0.01	2.80 ± 0.15	0.20 ± 0.05
60	0.75 ± 0.08	0.94 ± 0.04	2.85 ± 0.85	0.65 ± 0.35
80	0.77 ± 0.04	0.95 ± 0.06	3.10 ± 0.40	0.25 ± 0.10

Errors are given as standard deviations

Concurrent results were reported by Varanda et al. (2000). Analogously, the calcium ion concentration does not affect values of the Hurst exponent significantly (Barbosa et al. 2007). This is consistent with our modelling approach in which we neglect the details of operation of the gating sensors.

### Analysis of the generated time series

From both of our models we generated *n* = 5 time series with *N* = 6 × 10^6^ samples for five different values of the variable responsible for sensor–gate coupling. For each of the time series, we evaluated the open state probability (*p*_o_) and mean open and closed dwell times; we then performed Hurst analysis of the corresponding dwell-time series of subsequent openings and closings. To determine whether the long-range memory found for the simulated data is its intrinsic feature, the simulated data were shuffled, and the Hurst exponent was calculated for a randomized time series. Table 4 shows the values of the drift-regulating term, the mean values of open state probability, mean open and closed dwell times, the Hurst exponent corresponding to generated time series *H*, and the Hurst exponent obtained after shuffling (*H*_sh_).

**Table 4:** The values of characteristics arising from the gating stimulus, and the mean values and standard deviations (SD) of open state probability (*p*_o_)

*F*_d_ (kT/rcu)	*p*_o_ ± SD	*H* ± SD	*Τ*_open_ ± Δ*Τ*_open_ (ms)	*Τ*_closed_ ± Δ*Τ*_closed_ (ms)	*H*_sh_ ± SD
Model 1					
0.40	0.15 ± 0.01	0.74 ± 0.02	0.32 ± 0.01	1.75 ± 0.15	0.51 ± 0.01
0.20	0.25 ± 0.01	0.79 ± 0.01	0.46 ± 0.02	1.38 ± 0.11	0.52 ± 0.01
0.00	0.50 ± 0.01	0.82 ± 0.01	0.74 ± 0.07	0.74 ± 0.07	0.52 ± 0.01
−0.20	0.74 ± 0.01	0.79 ± 0.01	1.25 ± 0.09	0.43 ± 0.20	0.51 ± 0.01
−0.40	0.85 ± 0.01	0.73 ± 0.01	1.62 ± 0.04	0.31 ± 0.01	0.53 ± 0.01

TP	*p*_o_ ± SD	*H* ± SD	*Τ*_open_ ± Δ*Τ*_open_ (ms)	*Τ*_closed_ ± Δ*Τ*_closed_ (ms)	*H*_sh_ ± SD
Model 2					
14	0.16 ± 0.02	0.69 ± 0.01	0.63 ± 0.01	3.25 ± 0.44	0.51 ± 0.01
7	0.32 ± 0.02	0.71 ± 0.01	1.35 ± 0.08	2.93 ± 0.46	0.53 ± 0.01
0	0.50 ± 0.01	0.72 ± 0.02	2.16 ± 0.28	2.13 ± 0.25	0.52 ± 0.01
−7	0.68 ± 0.02	0.71 ± 0.01	2.87 ± 0.45	1.32 ± 0.08	0.52 ± 0.01
−14	0.85 ± 0.02	0.68 ± 0.01	3.79 ± 0.66	0.64 ± 0.02	0.51 ± 0.01

Hurst exponents: *H* for original, and *H*_sh_ for shuffled simulated time series. The aforesaid values were calculated as means from five time series (Model 1, Model 2) for each stimulus-regulatory value

The mean *r* ^2^ coefficient of linear regression used to evaluate *H* for all dwell time series generated by Model 1 was equal to 0.994 (0.996 for the corresponding *H*_sh_) and for those generated by Model 2—0.987 (0.995 for the corresponding *H*_sh_).

The values obtained indicate that our models enable estimation of approximate ionic current characteristics in quite good agreement with those obtained experimentally under different external conditions. By tuning the regulatory variables *F*_d_ (Model 1) or TP (Model 2) one obtains different open probabilities in the generated time series, which mimic the behaviour of the experimental time series under different gate-activating conditions (e.g. membrane depolarization).

**Fig. 10:**
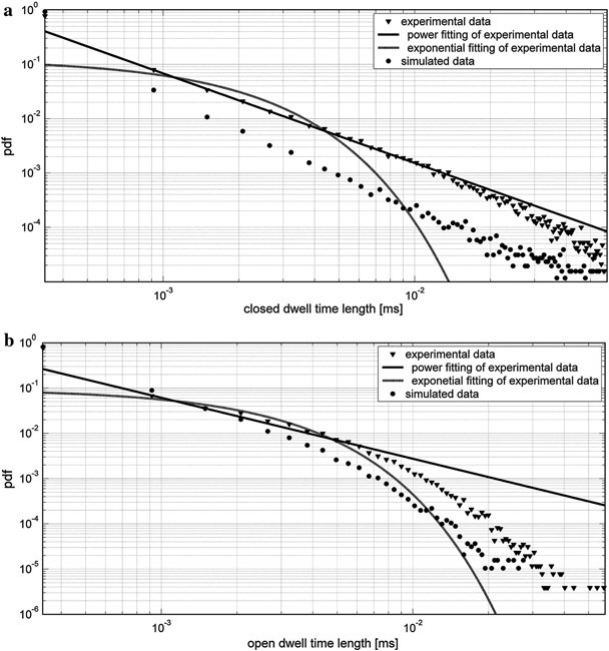
Closed (**a**) and open (**b**) residence time distribution for simulated time by use of Model 1, and recorded experimentally at [Ca^2+^] = 2 mM and *V* = 80 mV. Simulation data are given in Table 1, and the drift coefficient was chosen appropriately to obtain the open state probability possibly close to experimental data. The time unit for modelled data was rescaled to the experimental value. The fitting procedure was performed for experimental data, first, in linear coordinates; the results were then transformed to the logarithmic scale

**Fig. 11:**
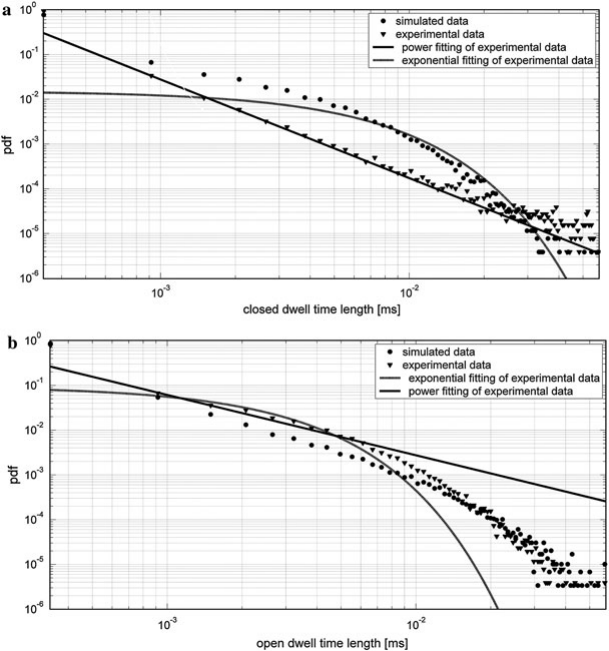
Closed (**a**) and open (**b**) residence time distribution for simulated time by use of Model 2, and recorded experimentally at [Ca^2+^] = 2 mM and *V* = 80 mV. Simulation data are given in Table 2, and the threshold point was chosen appropriately to obtain the open state probability possibly close to experimental data. The time unit for modelled data was rescaled to the experimental value. The fitting procedure was performed for experimental data, first, in linear coordinates; the results were then transformed to the logarithmic scale

The mean open dwell times generated by Model 2 are in quantitative agreement with experimental values, and the opening probability grows with open interval length, as expected. This tendency is conserved at a qualitative level in the data generated by Model 1. The long-term memory in the simulated data is exhibited independently of *p*_o_ (as expected), and the values obtained for the Hurst exponent are close to those corresponding to the experimental open and closed dwell time series. After a shuffling procedure, the long-term memory has disappeared (*H*_sh_ ≈ 0.5), which means that it is an inherent property of the considered system. The open and closed state dwell-time distributions obtained for the time series generated by Models 1 and 2 were plotted on a log–log scale in Figs. 10 and 11, respectively, and compared with the experimental distribution.

As follows from Fig. 10 Model 1 generates a power law dependence in the closed dwell-time distribution. This result coincides qualitatively with the empirical data and the literature suggestions that this kind of dependence should be expected at least in some cases (Goychuk and Hänggi 2002, 2003). In contrast, open dwell time distribution has an exponential tail. In both cases short dwell times are overestimated, and long dwell times are underestimated in comparison with experimental data.

Model 2 enables reconstruction of a correct open dwell time distribution, but the closed dwell time distribution is no longer power law, and is characterized by an exponential tail.

Values of the potential barrier energy separating open and closed states (*U*_TP_ − *U*_TP−1.5_) equal to 1.0 kT (Model 1) and 0.2 kT (Model 2), and the diffusion coefficient of the density fluctuations equal to 600 (Model 1) and 1,200 (Model 2) times the diffusion coefficient of reaction coordinate were set to generate a time series which gave the current characteristics similar to the experimental characteristics and had long-term memory as observed in the measurements. The assumed height of the activation barrier is not very large, which concurs with the diffusive nature of the gate dynamics, in which a rather smooth transition from open to closed conformation is expected. Moreover, the energy structure of the conformational space expressed by a smooth function without significantly differing peaks looks quite reasonable from a biological viewpoint, taking into account the existence of a large number of similar protein allosteric states with similar energy.

## Conclusion and outlook

In this work we have proposed two random-walk models of BK channel gate dynamics with long-term memory. Their construction was motivated by Hurst analysis applied to the experimental data measured in the voltage range from −80 to 80 mV and for a Ca^2+^ concentration of approximately 2 mM, supported by the results obtained by other authors under different experimental conditions (Bandeira et al. 2008; Barbosa et al. 2007; Varanda et al. 2000), which suggest that intrinsic long-term memory exists in the series of subsequent single channel state dwell-times.

According to the experiments, the mechanism responsible for the long-term memory seems not to be directly related to the voltage and calcium sensors’ activation (but may, still, be related to their activity). Taking into account the biological context of the BK channel system, it is reasonable to assume that thermal fluctuations of the activation gate machinery by itself could be responsible for the spontaneous transitions between the open and closed states of the channel under fixed conditions of the activating voltage and [Ca^2+^]. Nevertheless, the simple random fluctuations of an activation gate are not enough to produce the long-term memory, and a degree of synchronicity is required.

We have shown how to reproduce the long-term correlations in the gate dynamics by membrane thickness fluctuations, which affect the available size of conformational space and/or induce stress on the gating segments. The slow thermal fluctuation of the membrane density surrounding the activation gate was incorporated by introduction of the synchronous diffusion space boundary fluctuations in Model 1, and the drift force fluctuations in Model 2. The synchronicity is thought to originate from a channel reaction to squeezing or stretching. For example, if the cell membrane underlies squeezing, a part of the membrane (here: the channel’s surrounding) increases its density. This also affects the channel gate by narrowing the distribution of its conformational states.

The part of the channel protein responsible for channel gating has notably smaller mass than the whole channel protein immersed in the cell membrane. This difference manifests itself in the fluctuation time scales of the channel gate and its surrounding. To illustrate a connection between the physical and theoretical properties of the channel system consider the following reasoning:

Within a random walk framework, the diffusion coefficient *D* can be described by the formula:

17 $$ D \propto v \cdot \delta = v^{2} \cdot \tau $$

where *v* is the velocity of a considered object, *δ* denotes the mean free path, and *τ* is the mean time between subsequent collisions with the environment.

The frequency of collisions is proportional to the object’s surface (*S*), so *τ* is inversely proportional to S. Taking this into account, and the proportionality between the mass and volume (*m* ~ *V* ~ *S* ^3/2^), the following relationship is valid:

18 $$ \tau \propto m^{ - 2/3} . $$

According to the equipartition theorem:

19 $$ kT \propto \frac{1}{2}mv^{2} , $$

where *k* is the Boltzmann constant and *T* is temperature.

By combining Eqs. (17)–(19), one obtains the relationship between the mass and diffusion coefficient:

20 $$ m \propto \left( \frac{2kT}{D} \right)^{3/5} . $$

Thus, the relationship between the diffusion coefficients (and time scales) used in our models corresponds to the ratio of the masses of the considered channel elements:

21 $$ \frac{{m_{\text{S}} }}{{m_{\text{CG}} }} = \left( {\frac{{D_{\text{CG}} }}{{D_{\text{S}} }}} \right)^{3/5} $$

(subscripts: S, “surrounding”; CG, channel gate).

The models predict a ratio between the gate’s diffusion coefficient and diffusion coefficient of the surroundings expressed by $\frac{{D_{\text{S}} }}{{D_{\text{CG}} }} = \frac{{D_{\text{RC}} }}{{D_{\text{B}} }} = 600$ and $\frac{{D_{\text{S}} }}{{D_{\text{CG}} }} = \frac{{D_{\text{RC}} }}{{D_{\text{DF}} }} =$ 1,200 for Model 1 and Model 2, respectively. The problem, however, is that such a gate diffusion coefficient may not necessarily be the “true” gate diffusion coefficient, and it may be an apparent one observed because of a low sampling rate in a self-similar gating process (Liebovitch and Toth 1990).

Considering the S6-helix of a BK channel of *r* = 2.5 Å radius, its diffusion coefficient in the cell membrane should take a value similar to *D* ≈ 4 × 10^−8^ cm^2^/s = 4 × 10^−12^ m^2^/s (Almeida and Vaz 1995). To estimate our apparent gating diffusion coefficient we need to approximate our reaction coordinate unit (rcu) by some physical quantity. It seems reasonable to assume that the S6-helix may function as the channel gate. The channel vestibule diameter is approximately equal to *d* ≈ 20 Å (Cui et al. 2008). From the symmetry of the BK channel system (which is a tetrameric protein) one S6-helix has a *d*/2 = 10 Å range of movements. In our simulation, this distance is projected on a lattice of length equal to 2*B*_MAX_ length (Model 1). Thus:

22 $$ 1\,{\text{rcu}} \approx \frac{d}{{2 \times 2B_{{{\text{MAX}}}} }} = \delta  = 0.25\,{\AA} $$

i.e. 1 rcu corresponds to 0.25 Å.

The simulation time step length is equal to *τ* = 5 × 10^−5^ s (because of the experimental sampling frequency of 20 kHz), which renders the gate diffusion coefficient *D*_CG_ estimate:

23 $$ D_{\text{CG}} = \frac{{\delta^{2} }}{6\tau } = 1.04 \times 10^{ - 17} \left[ {\frac{{m^{2} }}{s}} \right]. $$

This value differs by six orders or magnitude from the expected value of *D*_CG_. This difference arises from the *τ* value restricted by the sampling frequency. Although more rapid channel gate fluctuations are not recorded, one cannot preclude their existence. According to Liebovitch and Toth (1990), the kinetic properties of patch-clamp recordings may have a self-similar nature. So, it is justified to consider the *D*_CG_ as an apparent diffusion coefficient resulting from the observation time scale *τ*, which should be higher (lower *τ*, fixed *δ* in Eq. (23)) on the thermal fluctuation scale.

The diffusion coefficient of the channel’s surrounding *D*_S_ was assumed in the simulation to satisfy Eq. (4) (Model 1), thus *D*_S_ may be estimated as *D*_CG_/600 = 1.73 × 10^−20^ $\left[ {\frac{{m^{2} }}{s}} \right].$ From Eq. (21), we can estimate the relationship between the actual mass of the fluctuating membrane (*m*_m_) and the channel gate (*m*), which is:

24 $$ \frac{{m_{\text{m}} }}{m} = \left( {\frac{D}{{D_{\text{S}} }}} \right)^{3/5} = 1.04 \times 10^{5} $$

To check whether this large ratio is acceptable physically, one may estimate the volume of the membrane patch used in the patch-clamp experiment, which could act as a “fluctuating surrounding” of the assumed channel gate. The volume of an S6-helix *V* (channel gate) may be approximated as a cylindrical volume spanning the membrane, which is:

25 $$ V = \pi \cdot r^{2} \cdot d_{\text{m}} \approx \pi \cdot (2.5)^{2} \cdot 70\,{\text{\AA}}^{ 3} = { 1,374}\,{\text{\AA}}^{ 3} $$

where *d*_m_ = 70 Å, is the average membrane thickness.

The volume of the whole-cell membrane patch *V*_wm_ is described as:

26 $$ V_{\text{wm}} = S_{\text{wm}} \cdot d_{\text{m}} = 10^{8} \cdot 70\, {\text{\AA}}^{ 3} = { 7} \times 10^{ 9} \,{\text{\AA}}^{ 3} $$

where *S*_wm_ denotes the surface area of the whole cell membrane patch, *S*_wm_ = 10^8^ Å^2^ (Sakmann and Neher 1995).

The ratio of volumes yields *V*_wm_/*V* = 5.09 × 10^6^. By use of this result, and the ratio of masses *m*_m_/*m* = 1.04 × 10^5^, one can infer that 2 % of the whole patch volume should be sufficient to act as the fluctuating “channel surrounding”. Such a result seems reasonable, and it may confirm, in some sense, the validity of our models.

The models can be further verified experimentally by measuring the membrane thickness fluctuations (possibly by changes in the membrane capacitance) simultaneously with the current recordings. In such an experiment, our model predicts a correlation between membrane thickness and local dwell time. A positive correlation between membrane thickness and dwell time would indicate Model 1, whereas a negative correlation would indicate Model 2.

Table 4 shows that the characteristics of the generated time series remain in a good agreement with those obtained experimentally (Table 3), in particular:

1. Our models enable generation of time series with different open state probabilities, and mean open dwell times which are in reasonable agreement with experimental values, recorded under different conditions of channel activating stimuli.
2. The Hurst analysis applied to the sequence of the opening and closing times in the simulated data furnishes mean values of 0.77 and 0.70 for Model 1, and Model 2, respectively. This result of the rescaled range analysis suggest that investigated data are long-term correlated, and the exponent remains in the allowed error range for the data (±0.1) (Hurst et al. 1965).

The Hurst exponents for the shuffled series show no long memory effects. In consequence, it is reasonable to assume that the trend-reinforcing behaviour is an inherent feature of the modelled systems.

Considering the experimental data obtained under the conditions described in the “Electrophysiology” section, it can be stated that the closed dwell-time distributions have power law dependence, and the open dwell-time distributions are characterized by an exponential tail. By use of Model 1 one can obtain the distributions which qualitatively reflect the empirical dependencies. By use of Model 2, qualitative and quantitative agreement in the open dwell time distributions is reached, but the experimental and simulated closed dwell time distribution types do not match exactly.

Details of the voltage and of the calcium sensors’ behaviour, and their effect on channel gate activity, were reduced in our models. The coupling between sensors and the gate was introduced in the simplest way possible: a constant drift acting on the reaction coordinate (Model 1) or a moveable threshold position (Model 2). As a consequence, our models provide only an approximate description of the dependence of gating on [Ca^2+^] and voltage, but the ideas presented can be introduced to more sophisticated models, for example MWC or HCA; this will, however, substantially increase their complexity.

## Figures and Tables

**Fig. 12:**
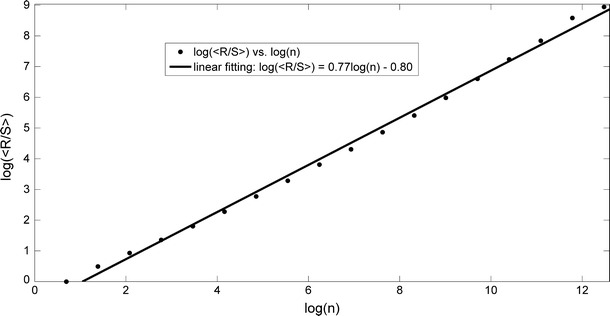
The Hurst exponent value (*H*) may be obtained by plotting Eq. (28) and evaluating its slope. In the figure, *H* is equal to 0.77

**Fig. 13:**
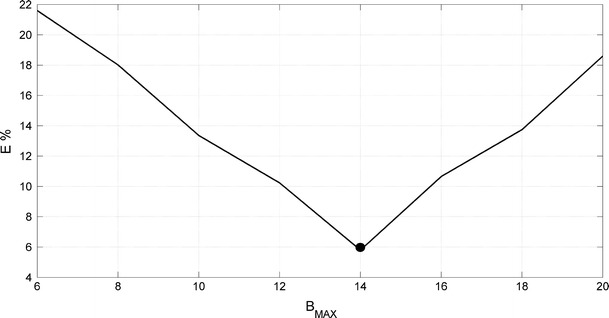
The total error as a function of *B*_MAX_ (Model 1). The values of all other variables are given in Table 1

**Fig. 14:**
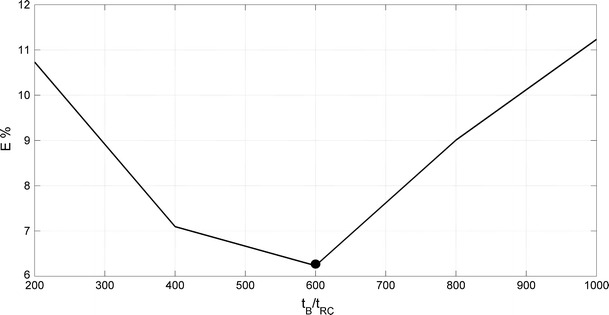
The total error as a function of the boundaries and reaction coordinate time scales ratio (Model 1). The values of all other variables are given in Table 1

**Fig. 15:**
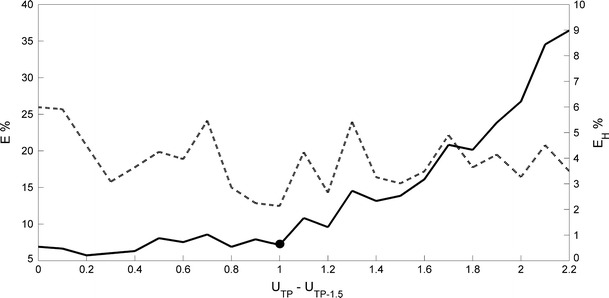
The total error (*E*) (values on the *left* *Y* axis) as a function the height of the potential barrier (Model 1). The appropriate error components corresponding to the Hurst exponent (*E*_H_) are represented by the *dashed line* (values on the *right* *Y* axis). The values of all other variables are given in Table 1

**Fig. 16:**
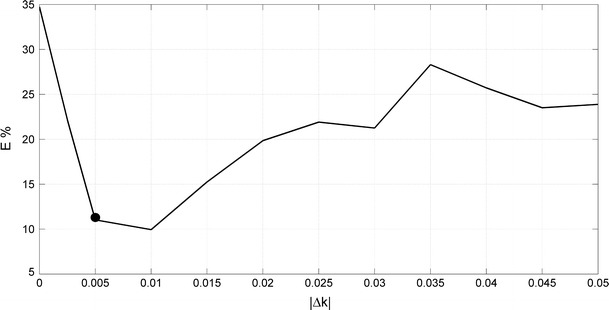
The total error as a function of the drift force’s increment |Δ*k*| (Model 2). The values of all other variables are given in Table 2

**Fig. 17:**
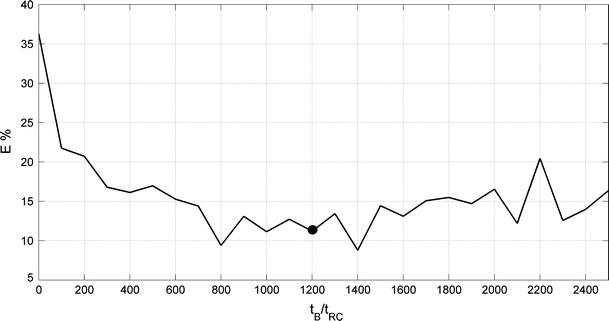
The total error as a function of the boundaries and reaction coordinate time scales’ ratio (Model 2). The values of all other variables are given in Table 2

**Fig. 18:**
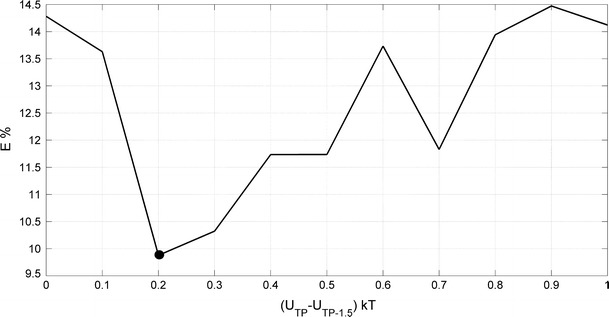
The total error as a function the height of the potential barrier (Model 2). The values of all other variables are given in Table 2

**Fig. 19:**
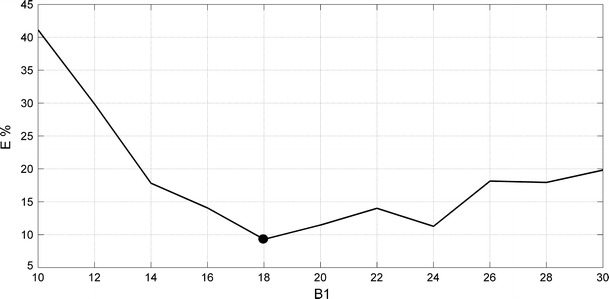
The total error as a function the boundary *B*1 location (Model 2). The values of all other variables are given in Table 2

## Appendix 1

Steps of the Hurst exponent evaluation procedure:

1. The given time series *T*_1_, *T*_2_,…,*T*_*N*_ of length *N* is divided into *d* subseries of *n* elements, such that $d \cdot n = N.$ The subseries obtained are denoted *I*_*m*_ (*m* = 1,…,*d*) and their elements as *t*_*m,k*_ (*k* = 1,…,*n*).
2. The mean value *E*_*m*_ and the standard deviation *S*_*m*_ for each subseries *I*_*m*_ are evaluated.
3. For each *I*_*m*_ an appropriate mean adjusted series *X*_*m*_ = *x*_*m,1*_,…,*x*_*m,d*_ is found, in which the elements are defined as *x*_*m,k*_ = *t*_*m,k*_ − *E*_*m*_.
4. The cumulative deviate series *Y*_*m*_ of elements $y_{m,k} = \sum\nolimits_{j = 1}^{k} {x_{m,k} }$are calculated.
5. The ranges *R*_*m*_ described as: *R*_*m*_ = max(*Y*_*1,m*_,…,*Y*_*n,m*_) *−* min(*Y*_*1,m*_,…,*Y*_*n,m*_) are found.
6. The rescaled range $\frac{{R_{m} }}{{S_{m} }}$ for each of the subseries is calculated. Then, the mean value $\left\langle {\frac{{R_{m} }}{{S_{m} }}} \right\rangle$ for the current division of the original time series is evaluated.

All aforementioned steps are then repeated for different lengths (*n*) of subseries. (What is often seen in the literature is that the lengths of subseries are chosen to be equal to the powers of 2 (*n* = 2^*p*^, where *p* = 1, 2, 3,…)).

The resulting relationship between $\left\langle {\frac{{R_{m} }}{{S_{m} }}} \right\rangle$ and *n* is described by power law scaling of the form:

27 $$ \left\langle {\frac{{R_{m} }}{{S_{m} }}} \right\rangle_{n} = c \cdot n^{H} $$

where *H* denotes the Hurst exponent and *c* is a constant.

Taking the logarithm from Eq. (27) one obtains:

28 $$ \log \left( {\left\langle {\frac{{R_{m} }}{{S_{m} }}} \right\rangle_{n} } \right) = H \cdot \log (n) + \log (c) $$

The Hurst exponent may be evaluated by use of the least-squares linear regression method, and its value is equal to the slope of the plot of Eq. (28) as illustrated in Fig. 12.

When one time series is analysed the goodness of fit (*R* ^2^) may be used as the error of the estimated Hurst exponent value. However, in the case when one has at least a few repetitions of the experiment, e.g. a series of independent measurements carried out under the same conditions (in our studies these are the patch-clamp time series for five patches on the same voltage values) it is better to use a standard deviation from the mean value obtained for the repetitions as a measure of the Hurst coefficient error. A fitting error for one series of finite length could not resemble the properties of a set of analogous series, because of the possibility of over or underestimation, owing to the poorly averaged terms in the fitting at large *n* values which are likely to perturb randomly the linearity of the data.

## Appendix 2

The Model 1 simulation procedure:

1. The lattice with the maximum number of nodes equal to 2*B*_MAX_ is set.
2. The initial positions of the reaction coordinate *x* and the fluctuation boundaries *B*1 and *B*2 are set. The threshold point (TP) is equal to 0. The *B*1 and *B*2 are symmetrically located around the TP.
3. The potential function (*U*(*x*)) described by Eq. (7) is associated with the lattice.
4. The position of the reaction coordinate is randomly changed for one step length in one time step, with the probability of movement to the right *p* and to the left *q* described by Eqs. (5) and (6), respectively.
5. The position of the reaction coordinate relative to the TP is checked. If the RC is at the right-hand side of the TP, the open state is recognized. Otherwise the closed state is stated.
6. If the RC reaches the *B*1 or *B*2 positions during its random walk, it is reflected to its previous position.
7. Steps 4, 5, 6 are repeated for a number of time steps, which is determined by Eq. (4).
8. The boundaries *B*1 and *B*2 are randomly and synchronously moved for one step length toward or away from the TP with equal probability. (If *B*1 reaches *B*_MIN_ or TP − 1 positions, it is reflected to its previous position. Analogously, if *B*2 reaches *B*_MAX_ or TP + 1 positions, it is reflected to its previous position.)
9. Steps 4–8 are repeated for a desired time series length.

The Model 2 simulation procedure:

1. The lattice with the maximum number of nodes equal to 2*B*2 and the position of the threshold point TP are set.
2. The initial position of the reaction coordinate *x* is set.
3. The potential function (*U*(*x*)) described by Eq. (15) is associated with the lattice. The initial “synchronous” drift is set to be equal to 0.
4. The position of the reaction coordinate is randomly changed for one step length in one time step with the probability of movement toward the TP (*p*_T_) and away from it (*q*_T_) described by Eqs. (5) and (6).
5. The position of the reaction coordinate relative to the TP is checked. If the RC is at the right-hand side of the TP, the open state is recognized. Otherwise the closed state is stated.
6. If the RC reaches the *B*1 or *B*2 positions during its random walk, it is reflected to its previous position.
7. Steps 4, 5, 6 are repeated for a number of time steps, which is determined by Eq. (4).
8. The potential slopes (and, as a consequence, the value of the synchronous drift force described by Eq. (16)) is randomly changed by a constant ±|Δ*k*| with equal probability.
9. Steps 4–8 are repeated for a desired time series length.

## Appendix 3

The model variables are determined by use of the gradient optimization technique. Optimization was performed until the sum of relative errors is less than 10 %. The values chosen are provided in Tables 1 and 2.

To support the quality of the values found we provide the dependencies of the total error in the model variables when one of them is being changed and the rest are kept constant and equal to the optimum values (Figs. 13, 14, 15, 16, 17, 18, 19). The error plots, being realizations of a stochastic process (they are evaluated after a simulation run), reveal some variability between realizations of the simulation, which can be characterized by a standard deviation of ±2 %.

On each of the figures, the chosen value is denoted by a dot.

From Fig. 15, it could be roughly stated that the total errors in the range of potential barriers from 0.2 to 1.0 kT maintain quite similar values. In the simulations, we have chosen its value equal to 1.0, because for lower values the error component corresponding to the Hurst exponent (*E*_H_) was relatively higher, as shown by the dashed line in Fig. 15. Minimizing the *E*_H_ is thought to be important, taking into account the main objectives of the model.

Considering the errors connected with |Δ*k*| estimation, although the total error was lower for |Δ*k*| = 0.010 than for |Δ*k*| = 0.005 (Fig. 16), we have regarded the second value as optimum, because of the corresponding Hurst exponent errors, which were equal to 0.066 and 0.053 for |Δ*k*| = 0.010 and 0.005, respectively.

Analysing the total error dependency on the time scales’ ratio (Fig. 17), one can state, that the ratios below 600 are characterized by relatively high total errors. In the range up to 1,800 one can observe errors of similar magnitude, which average to about 13 %. Then, for ratios larger than 1,800 the error grows again. Because the exact plot in range between 600 and 1,800 has variability depending on the simulation realization (it is a random variable), we have chosen the middle value of this interval, i.e. $\frac{600 + 1,800}{2} =$ 1,200.
